# Early neurodevelopmental outcome in newborns with mild hypoxic‐ischaemic encephalopathy

**DOI:** 10.1111/dmcn.70199

**Published:** 2026-02-03

**Authors:** Domenico M. Romeo, Chiara Arpaia, Angelo Di Malta, Elisa Pede, Grazia Maria Cicala, Daniela Ricci, Francesca Serrao, Eugenio Mercuri, Claudia Brogna, Mild Encephalopathy Regional Network*, Giovanni Vento, Chiara Cafforio, Eleonora Scapillati, Roberta Arena, Francesca Gallini, Vita Augusta, Andrea Guzzetta

**Affiliations:** ^1^ Pediatric Neurology Unit, Fondazione Policlinico A. Gemelli, IRCCS Rome Italy; ^2^ Pediatric Neurology Unit, Università Cattolica del Sacro Cuore Rome Italy; ^3^ National Centre of Services and Research for the Prevention of Blindness and Rehabilitation of Visually Impaired, IAPB Italia Onlus Rome Italy; ^4^ Neonatal Intensive Care Unit, Fondazione Policlinico A. Gemelli, IRCCS Rome Italy

## Abstract

**Aim:**

To describe the natural history of newborn children with mild hypoxic‐ischaemic encephalopathy in the first year of life.

**Method:**

This was a multicentre, prospective observational study involving five neonatal intensive care units in an Italian region using both structured clinical and neurophysiological assessments according to the Italian Society of Neonatology guidelines. To make the clinical evaluation homogeneous at each centre, a training neurological courses was provided to all operators involved in the study. Forty newborn children (23 males and 17 females) underwent neonatal and infant neurological examination (Hammersmith Neonatal Neurological Examination and Hammersmith Infant Neurological Examination), brain magnetic resonance at 7 to 10 days, electroencephalogram between 6 months and 12 months, and Griffiths Scales of Child Development, Third Edition to assess neurodevelopmental outcome at 12 months.

**Results:**

All of these infants demonstrated typical neuromotor development and age‐appropriate developmental quotients at 12 months, even in the few showing abnormalities at magnetic resonance imaging assessment or neurological assessment during the neonatal period.

**Interpretation:**

Current classifications of mild hypoxic‐ischaemic encephalopathy may misclassify risk, highlighting the need for refined diagnostic criteria and early staging tools that can be used within the first 6 hours of life.

## Abstract 2

**This original article is commented on by Cheong on pages** 1180–1181 **of this issue.**

**Abbreviations**
aEEG: amplitude‐integrated electroencephalogram
HIE: hypoxic‐ischaemic encephalopathy
HINE: Hammersmith Infant Neurological Examination
HNNE: Hammersmith Neonatal Neurological Examination

**What this paper adds**

- Mild hypoxic‐ischaemic encephalopathy (HIE) with normal amplitude‐integrated electroencephalogram and no seizures was reported at 12 months.
- Even infants with mild HIE abnormalities at magnetic resonance imaging or neurological assessment during the neonatal period reported favourable early outcomes.

Hypoxic‐ischaemic encephalopathy (HIE) due to perinatal asphyxia is the most common form of neonatal encephalopathy in infants born at term.^1^, ^2^ Neonatal encephalopathy was once automatically ascribed to hypoxia‐ischaemia; now we known that hypoxia‐ischaemia is only one of many possible contributors to neonatal encephalopathy, which requires specific evidence of hypoxia‐ischaemia such as sentinel events (e.g. uterine rupture), metabolic acidosis, organ injury, and suggestive findings on magnetic resonance imaging (MRI). However, not all newborn children who have obvious signs of encephalopathy exhibit clear signs or a history of perinatal asphyxia, suggesting that other variables, including antenatal factors, may contribute. This is true when we consider the incidence; neonatal encephalopathy is estimated to affect 2 to 6 per 1000 term births, of which HIE accounts for approximately 1.5 per 1000 term births.

Because different patterns of evolution are related to HIE, specific clinical features of the neonatal neurological assessment are particularly useful for prognostic evaluation, that is, clinical severity, the presence and timing of seizure onset, and the duration of neurological abnormalities. Outcome is generally related to clinical grading of severity, with children with moderate and severe grades having the highest rates of neurodevelopmental disabilities and death.^1^, ^2^

Although it is unclear whether seizures independently exacerbate brain injury or merely reflect more extensive cerebral damage, their occurrence is associated with a twofold to fivefold increase in the risk of sequelae,^1^, ^2^ particularly when seizures manifest early. Regarding symptom duration, clinical experience suggests that neonates who exhibit neurological signs lasting less than 1 to 2 weeks have a favourable prognosis for typical early childhood development, although data on school‐age outcomes are limited.^3^, ^4^, ^5^

Beyond clinical examination, instrumental assessments could provide further valuable prognostic information. Electroencephalogram (EEG) patterns and their temporal evolution represent a particularly useful bedside tool. However, amplitude‐integrated EEG (aEEG) is most commonly used in neonatal intensive care units under neonatologist supervision and using simplified configurations, typically involving an interhemispheric lead (P3–P4) or two central‐parietal leads (C3–P3, C4–P4). This reduced‐channel aEEG/EEG set‐up is known as cerebral function monitoring.^6^, ^7^, ^8^, ^9^, ^10^, ^11^, ^12^ Many studies have highlighted the utility of cerebral function monitoring for brain monitoring in neurologically at‐risk neonates,^6^ including those with acute encephalopathy undergoing hypothermic treatment,^7^ experiencing epileptic seizures,^8^ with cardiac malformations in the perioperative period,^9^ high‐risk infants born preterm,^10^ and cases complicated by brain lesions such as intraventricular haemorrhage.^11^

The introduction of hypothermia as a neuroprotective treatment has significantly improved the prognosis of HIE. Hypothermia reduces the risk of disability, including cerebral palsy, cognitive impairment, learning disabilities, and epilepsy, in infants with moderate or severe HIE. It has led to a statistically significant and clinically meaningful reduction in the combined outcome of mortality and major neurodevelopmental disability. Moreover, hypothermia reduces abnormal general movements, which are early markers of neurodevelopmental impairment.^13^, ^14^, ^15^

In 2023, the Italian Society of Neonatology released updated national guidelines that provide detailed recommendations for the use of either cerebral function monitoring or conventional EEG to support clinical decisions, particularly regarding the initiation of hypothermia in neonates with HIE.^16^ These guidelines emphasize timely identification of eligible infants (within 6 hours of life), based on a combination of gestational age, clinical criteria, acid–base status, neurological signs, and neurophysiological findings. The recommended scenarios for initiating hypothermia are summarized in Table 1.

**TABLE 1:** Summary of strong recommendations for initiating therapeutic hypothermia (Italian Society of Neurology 2023 Guidelines).

Strong recommendation	Clinical setting 1	Clinical setting 2
Basic criteria	Gestational age ≥ 35 weeks and birthweight >1800 g and age <6 hours	Gestational age ≥ 35 weeks and birthweight >1800 g and age <6 hours
Criterion A: peripartum asphyxia	pH ≤7.00 or base excess ≥ 12 mmol/l or Apgar score ≤5 at 10 minutes or need for respiratory support at 10 minutes	pH ≤7.00 or base excess ≥ 12 mmol/l or Apgar score ≤5 at 10 minutes or need for respiratory support at 10 minutes
Criterion B: neurological signs	At least two abnormal neurological signs present (from predefined list)	At least two abnormal neurological signs present (from predefined list)
Criterion C: EEG or aEEG findings	aEEG or EEG showing moderate or severe abnormalities	Criterion not evaluable^a^

^a^ Not evaluable because of unavailability of amplitude‐integrated electroencephalogram (aEEG) or electroencephalogram (EEG).

Currently, over 50% of neonates diagnosed with HIE are classified as having mild HIE and do not meet criteria for therapeutic hypothermia.^17^, ^18^, ^19^, ^20^ These infants are generally considered at low risk for adverse outcomes and consequently are often excluded from follow‐up programmes and specific interventions. However, recent evidence indicates that mild HIE carries an increased risk of brain injury, with a high incidence of abnormalities detected on brain MRI and a broad spectrum of potential neurodevelopmental complications, including learning disabilities, neuropsychological disorders, epilepsy, visual impairments, and sensory deficits.^17^, ^18^, ^19^, ^20^ Recently, the PRIME study reported important knowledge gaps regarding the prospective outcomes of infants with mild HIE diagnosed at less than 6 hours of life with a contemporary follow‐up at 18 to 22 months of age,^18^, ^20^ reporting neurodevelopmental disabilities between 16% and 40%. These findings underscore the emerging recognition that the outcomes of newborn children outside cooling criteria and with mild grading is not uniformly favourable. However, specific knowledge gaps should still be considered: lack of a systematic definition of mild HIE; lack of precise early clinical and instrumental signs that can predict which infant with mild HIE will develop moderate or severe disabilities; and lack of neurodevelopmental outcome studies in infants with mild HIE.

Therefore, the aim of the present study was to determine the early natural history of infants with an early diagnosis (≤6 hours of age) of mild neonatal encephalopathy (HIE) who do not qualify for therapeutic hypothermia, using both structured clinical and neurophysiological assessments.

## METHOD

### Study population

This multicentre, prospective observational study included neonates born between 2020 and 2023 from four intensive neonatal intensive care units in the Lazio region of Italy, each contributing different clinical and research expertise. To minimize subjective bias, one or two clinicians from each centre underwent standardized neurological training to harmonize the assessment and reach the best interobserver agreement for the neurological assessments of newborn children and infants. The training courses were conducted by paediatric neurologists who are experts in the neurological assessment of newborn children and infants at low and high risk (EM, DR, DMR, AG).

After the training course, each neonatal intensive care unit started the recruitment of newborn children with an early diagnosis (≤6 hours of age) of mild HIE who were did not qualify for therapeutic hypothermia.

In this study, infants fulfilling all established diagnostic criteria for HIE, including clinical signs, biochemical evidence, or perinatal sentinel events were classified as having HIE, while infants with neonatal encephalopathy due to other causes were excluded.^1^, ^2^

Inclusion criteria were newborn children born at a gestational age of at least 36 weeks and presenting with neurological signs of encephalopathy observed between 10 minutes and 6 hours after birth. In addition, at least one indicator of intrapartum hypoxia had to be present. These indicators included biochemical evidence, such as an arterial or cord blood pH equal to or below 7.0, or a base excess of 12 mmol/l or more within the first hour of life. Relevant perinatal events suggestive of hypoxia were also considered, including abnormal fetal heart rate patterns, cord prolapse, uterine rupture, maternal haemorrhage, trauma, maternal seizures, cardiopulmonary arrest, shoulder dystocia, meconium‐stained amniotic fluid, or a prolonged second stage of labour. A low Apgar score (≤5 at 10 minutes) or the need for continuous respiratory support at 10 minutes of life was also accepted as indicative of perinatal hypoxia.

Infants who fulfilled these criteria (criterion A: peripartum asphyxia) underwent an aEEG and a standardized neurological examination using the modified Sarnat score within the first 6 hours of life.^18^, ^20^ The score evaluated six categories: level of consciousness; spontaneous activity; posture; tone; primitive reflexes (suck and Moro); and autonomic nervous system (pupils, heart rate, and respiration). Each category was scored for predefined signs consistent with normal, mild, moderate, or severe HIE.

Mild HIE (Sarnat stage 1 or Sarnat stages 1–2) was defined according to: (1) the modified Sarnat score, as the presence of mild abnormality (score ≥1) in at least one of the six assessed neurological domains (level of consciousness, spontaneous activity, posture, tone, primitive reflexes, autonomic function) in infants who did not meet the criteria for therapeutic hypothermia, that is, with no evidence of moderate or severe HIE (defined as moderate or severe abnormalities in three or more neurological domains), corresponding to Sarnat 2 and Sarnat 3 as described previously;^18^, ^20^ and (2) a normal aEEG, defined as continuous normal voltage (maximum voltage 10–50 μV and minimum voltage 5–10 μV).^18^, ^20^ Sarnat stages 1 to 2 are often used to describe newborn children transitioning between mild (stage 1) and moderate (stage 2) encephalopathy, showing signs that bridge the gap between mild and moderate encephalopathy.

Exclusion criteria included the absence of neurological abnormalities, failure to enrol the patients within the first 6 hours of life, presence of major congenital anomalies or cerebral malformations, a diagnosis of neonatal abstinence syndrome, metabolic encephalopathy, birthweight equal to or below 1800 g, treatment with therapeutic hypothermia, or lack of informed parental consent.

### Clinical and instrumental assessments

All newborn children had clinical and instrumental assessments performed during the neonatal period: neurological examination using the modified Sarnat score^18^, ^20^ and the Hammersmith Neonatal Neurological Examination (HNNE) at 6 hours or less of life and before being discharged home;^21^ HNNE scores were interpreted using standardized optimality scores, with a total score below 30.5 indicating atypical neurological findings or suboptimal findings, while scores at or above this value were considered within the typical range or optimal;^22^, ^23^, ^24^, ^25^ aEEG within 6 hours and at 24 hours of age for at least 30 minutes; cranial ultrasound ≤6 hours of age and before discharge home; and brain MRI between 7 days and 10 days of age.

After discharge home, newborn children were involved in a follow‐up of at least 12 months with an early neurological outcome (3 months, 6 months, 12 months): the Hammersmith Infant Neurological Examination (HINE) at follow‐up and the General Movements Assessment.^22^, ^23^, ^24^, ^25^

The HINE total scores (range = 0–78) were calculated by summing individual item scores. Interpretation was based on validated age‐specific cut‐offs: lower scores indicate impaired neurological function, while higher scores reflect typical development.^22^, ^23^, ^24^

Neurodevelopmental outcome was assessed using the Griffiths Scales of Child Development, Third Edition. Developmental quotients were classified as normal (developmental quotient ≥ 85), borderline (developmental quotient = 70–84), or impaired (developmental quotient < 70), in accordance with the standardized criteria of the test manual (EEG between 6 months and 12 months).^26^

The study protocol was approved by the ethics committee of our institution (ID 2631, protocol no. 0000776/20; 9th January 2020); written informed consent was obtained from parents.

### Statistical analysis

The HNNE and HINE scores are reported as the mean, SD, and range at the different ages.

## RESULTS

### Study population and baseline characteristics

Forty newborn children (23 males and 17 females) were enrolled from five hospitals within the Lazio region of Italy. Specifically, 22 newborn children were recruited at the Policlinico Universitario Agostino Gemelli, 11 at the San Camillo Forlanini Hospital, four at the San Pietro Fatebenefratelli Hospital, and thee at the Fatebenefratelli Hospital‐Isola Tiberina. All completed the 12‐month follow‐up. All newborn children presented a normal aEEG within 6 hours and at 24 hours of age and no seizures during hospitalization. Forty per cent of newborn children had a normal cranial ultrasound at birth, while 54% presented with mild periventricular hyperechogenicities and 6% exhibited ventricular asymmetry. Before discharge home, 80% of newborn children had a normal cranial ultrasound, 14% showed deeper hyperechogenicities, and 6% exhibited ventricular asymmetry.

### Neonatal neurological assessment

Neurological status was assessed using the HNNE and the modified Sarnat score. The mean (SD) scores obtained with the HNNE at less than 6 hours and at hospital discharge (7–10 days of age) were 26 (3.04; range = 19–32) and 29 (2.83; range = 21–32) respectively; the optimality score (>30) according to the normative data^21^ was reached for five and 15 newborn children respectively.

**FIGURE 1:**
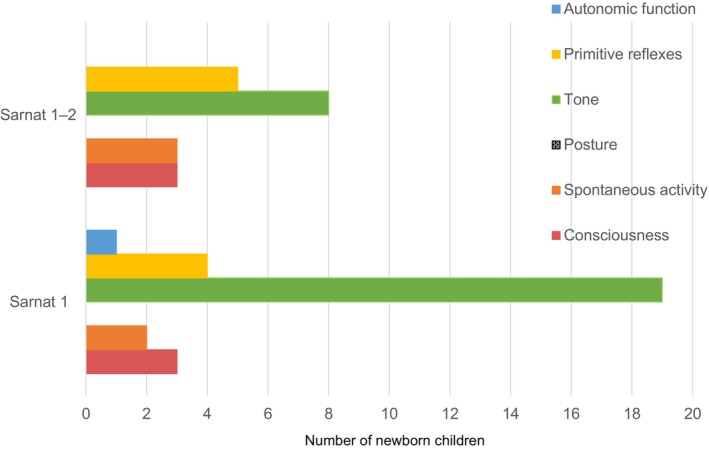
Sarnat score in the population.

At birth, 60% of newborn children were classified as Sarnat stage 1, indicating mild neurological abnormalities, while 40% presented with features falling between Sarnat stages 1 and 2 (Figure 1).

According to the modified Sarnat score and HNNE, hypotonia was the most frequently observed feature, often accompanied by transient alterations in consciousness. Reflex responses were generally preserved, although the Moro reflex was absent or incomplete in approximately 20% of children. Benign tremors were observed in about 30% of cases; when including perinatal startle responses, the prevalence rose to over 50%. Immature visual orientation was identified in 35% of infants, while signs of irritability or heightened vigilance were observed in approximately one‐quarter of infants. Less frequent findings included fragmented visual pursuit and challenges in consolability affecting around 10% of infants.

### Neuroimaging findings

Brain MRI performed between 7 days and 10 days of age revealed normal findings in 75% (*n* = 30) of newborn children. Mild abnormalities were detected in 17.5% (*n* = 7) of children and included punctate white matter lesions (<6 mm), diffuse excessive high signal intensity, and minor asymmetry of the lateral ventricles. In 7.5% (*n* = 3) of cases, infants showed more significant findings: one presented with multifocal microhaemorrhages involving the deep white matter and cerebellar hemispheres, while the other two had a unilateral focal ischaemic lesion in the putamen.

### Follow‐up neurological and neurodevelopmental outcomes

#### At 3 months

At 3 months of age, neurological assessment using the HINE revealed a mean score of 59 (range = 43–65), slightly below the normative reference values (mean = 65, SD 5.26, range = 62–69).^22^, ^25^ Tone items were specific items with low scores.

#### At 6 months

**FIGURE 2:**
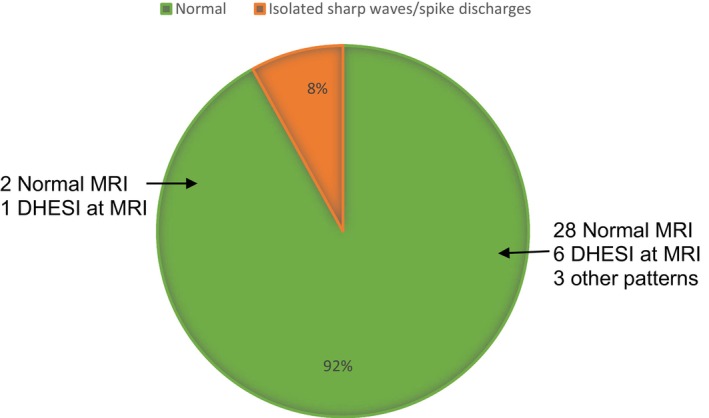
Electroencephalogram pattern. Abreviations: DHESI, diffuse excessive high signal intensity; MRI, magnetic resonance imaging.

At the 6‐month follow‐up, the mean (SD) HINE score increased to 69 (4.07; range = 65–72), aligning with the normative values (mean = 69, range = 64–74).^22^, ^25^ EEG recordings (between 6 months and 12 months of age) performed during both wakefulness and sleep revealed age‐appropriate organization of background activity, without evidence of epileptiform discharges in most infants with mild HIE and normal or mildly abnormal MRI findings. Three infants presented with isolated sharp waves or spike discharges in the occipital or frontotemporal regions; however, these findings were not associated with clinical seizures and no diagnosis of epilepsy was made (Figure 2).

#### At 12 months

At the 12‐month follow‐up, the mean (SD) HINE score increased to 74 (2.89; range = 66–78), which is in line with the reference data (mean = 74, range = 65–78).^22^, ^25^ No infant exhibited clinical signs consistent with cerebral palsy, including those with lower HINE scores at 3 months.

At 12 months of age, neurodevelopmental assessments using the Griffiths Mental Development Scales, Third Edition showed a score above average in most infants (60%), while 30% fell within the expected range for age; borderline scores were observed in 10% of the cohort. No infants scored clinically or significantly below normal. These last patients had normal neuroradiological patterns and HNNE and HINE scores.

### Relationship between neonatal neurological assessment and outcome

At the neonatal neurological evaluation, 29 newborn children (72.5%) reported one abnormal sign at the Sarnat score; hypotonia was the prevalent sign reported in 66% of cases; for these 29 newborn children, the HNNE scores were under the optimality value (< 30.5) in 62% of cases; 52% presented HINE scores lower than the reference range at 3 months; at 6 months and 12 months, 100% reported HINE scores within the reference range and normal quotient on the Griffiths Mental Development Scales, Third Edition at 12 months.

Eleven newborns (27.5%) in the cohort presented with two abnormalities during the early neurological assessments with the Sarnat score. These included altered consciousness, abnormal muscle tone, atypical spontaneous activity, and altered primitive reflexes. Specifically, six infants had an immature Moro reflex, nine presented with abnormal tone (six hypotonic and three with hyperextension of the head–trunk axis), four had reduced spontaneous motility, and three exhibited hypervigilance. All of these 11 newborn children presented a HNNE score under the optimality value (< 30.5); 82% presented HINE scores lower than the reference range at 3 months; at 6 months and 12 months, 100% reported HINE scores within the reference range and a normal quotient with the Griffiths Scales of Child Development, Third Edition at 12 months. Although some patients presented some abnormalities at MRI, all reported a typical outcome, without any additional neurological problems.

## DISCUSSION

This study evaluated short‐term neurological and developmental outcomes in neonates with mild HIE who did not receive therapeutic hypothermia. The results suggest that all of these infants demonstrated favourable early outcomes, including typical neuromotor development and age‐appropriate developmental quotients at 12 months, even in those showing abnormalities on MRI or at neurological assessment during the neonatal period.

These findings align with the emerging literature suggesting that many cases of mild HIE may follow a benign course. However, other studies reported a broader spectrum of neurodevelopmental outcomes, including language delay, attention deficits, and visual‐perceptual difficulties at school age.^17^, ^18^, ^19^, ^20^ A systematic review of 20 studies (totalling 341 infants with mild HIE) found that approximately 25% had atypical neurodevelopmental outcomes by 18 to 22 months, including death, cerebral palsy, or standardized test scores more than 1SD below the mean.^17^ Chalak et al.^20^ reported a 16% incidence of disability at 18 to 22 months, with 40% scoring below 85 in the cognitive, motor, or language domains using the Bayley Scales of Infant and Toddler Development, Third Edition.^20^ Notably, a large prospective cohort analysis demonstrated that at 2 years of age, infants with mild HIE scored significantly lower on cognitive composite scores (mean = 97.6 vs 103.6 in controls).^27^

A key challenge in the field remains the lack of a universally accepted definition for mild HIE and the frequent absence of standardized early evaluative clinical examination tools and staging in the first 6 hours of life despite the evolution of HIE over time.^17^, ^27^, ^28^, ^29^ This holds true when we consider a recent trial performing therapeutic hypothermia in children with mild HIE, which showed no specific improvements in newborn children treated versus not treated.^30^, ^31^ However, these studies reported some potential bias, including newborn infants with or without an abnormal EEG at baseline and with seizures, and also lacking specific clinical neurodevelopmental outcomes.

In the present study, newborn infants with mild HIE included those with subacute exposure to hypoxia‐ischaemia not reaching the criteria for therapeutic hypothermia according to the modified Sarnat score and according to the recent Italian guidelines for newborn children with HIE who are candidates for therapeutic hypothermia. All of these newborn infants showed a normal aEEG at both 6 hours or less and 24 hours of age. Therefore, our results were probably due to a strict definition of mild HIE, including those infants with a normal aEEG only performed at birth and at 24 hours of age with no seizures.

The main early clinical neurological feature of the present population was the presence of hypotonia and an immaturity of reflexes (e.g. Moro reflex), with suboptimal score at the HNNE at both evaluations (<6 hours and at discharge); however, these signs were typically transient, resolving within the first 3 to 6 months. Few infants continued to present axial hypotonia at 3 months, with a further improvement at follow‐up, with only 15% reporting suboptimal scores (< 73) at 12 months with the HINE, but within the reference data scores.^22^, ^23^, ^24^, ^25^ Furthermore, none of the infants scored under the cut‐off for infants at risk of or with clinical signs of cerebral palsy or neurodevelopmental disabilities.^22^, ^23^, ^24^, ^25^ Even those infants with more immature clinical signs at birth, reported a Griffiths Scales of Child Development, Third Edition score within the typical range, and only 10% reported a score in the borderline range, probably because of sociocultural or environmental factors. A subset of patients with mild and non‐specific findings on MRI (e.g. punctate white matter lesions, diffuse excessive high signal intensity, mild ventricular asymmetries) also demonstrated typical neurodevelopmental outcomes. Similarly, sporadic paroxysmal EEG findings did not correspond to clinical epilepsy or developmental delays.

All of the infants showing minimal immaturity in neurological or neurodevelopmental assessment at 12 months were reassessed for clinical proposal at 24 months; all showed a typical neurodevelopmental outcome.

These findings support the transient nature of some early neurological and neuroradiological signs in mild HIE and emphasize the importance of structured clinical and instrumental assessments for further studies in this population of infants. Current classifications may underestimate or misclassify risk, highlighting the need for refined diagnostic criteria and early staging tools that can be used within the first 6 hours of life. This variability complicates comparisons across studies and limits the applicability of findings.^31^ Although therapeutic hypothermia is not currently indicated for mild HIE, our results reinforce the importance of structured neurological and developmental follow‐up programmes in this population of infants.

A key finding of our study is related to the definition of mild HIE. By applying more stringent clinical and neurophysiological criteria, we identified a subgroup of infants with mild HIE in whom the higher rates of adverse neurodevelopmental outcomes reported in previous studies were not replicated. This suggests that the heterogeneity within the category of mild HIE may substantially influence reported outcomes; stricter inclusion criteria may select a population with a truly milder clinical course. From a clinical perspective, this approach may help clinicians to better stratify risk, identify infants at genuinely low risk of adverse outcomes, and tailor follow‐up strategies accordingly, while providing more accurate counselling to families. From a research standpoint, the use of more homogeneous and clearly defined criteria for mild HIE may improve comparability across studies and could help explain discrepancies in the literature, supporting more precise selection of infants for future observational studies and interventional trials.

This study has some limitations. The sample size was relatively small and limited to a single region (Lazio, Italy). Additionally, follow‐up was restricted to 12 months; subtle cognitive or behavioural impairments may emerge later, particularly at school age. Future research should aim to include larger multicentre cohorts and extend follow‐up into later childhood.

### Conclusion

To the best of our knowledge, the present study is the first to explore the early natural history of infants with mild HIE using structured clinical, neurophysiological, and neuroimaging assessments. These children with mild HIE could be defined as ‘at really low risk’ and probably not needing a follow‐up. We suggest using the criteria of a normal aEEG and no seizures to define low risk in mild HIE, which probably should not be included in further trials of therapeutic hypothermia in mild HIE. Because the criteria for therapeutic hypothermia should be reached within the first 6 hours, the assessment of aEEG could be prolonged to more than 30 minutes.

The use of more stringent criteria to define mild HIE may allow better risk stratification in clinical practice and improve the homogeneity and comparability of populations included in future research studies.

Future comparative studies assessing treated versus untreated infants with mild HIE would also help determine whether current management protocols adequately address this intermediate‐risk population. A promising avenue for future research would also be the collection of genomic material from these infants because emerging evidence suggests that some neonates may be more genetically susceptible to hypoxic insults during birth, thus facilitating the development of targeted therapies and contributing to morbidity reduction and prognosis.^32^

## Data Availability

The data that support the findings of this study are available on request from the corresponding author (DMR). The data are not publicly available due to privacy or ethical restrictions.

## References

[1] Shankaran S , Laptook AR , Ehrenkranz RA , Tyson JE , McDonald SA , Donovan EF et al. Whole‐body hypothermia for neonates with hypoxic‐ischemic encephalopathy. N Engl J Med 2005;353:1574–84. 16221780 10.1056/NEJMcps050929

[2] McIntyre S , Nelson KB , Mulkey SB , Lechpammer M , Molloy E , Badawi N ; Newborn Brain Society Guidelines and Publications Committee. Neonatal encephalopathy: Focus on epidemiology and underexplored aspects of etiology. Semin Fetal Neonatal Med 2021;26:101265. 34305025 10.1016/j.siny.2021.101265

[3] Shellhaas RA , Barks AK . Impact of amplitude‐integrated electroencephalograms on clinical care for neonates with seizures. Pediatr Neurol 2012;46:32–5. 22196488 10.1016/j.pediatrneurol.2011.11.004 PMC3246404

[4] Robertson CM , Finer NN , Grace MG . School performance of survivors of neonatal encephalopaty associated with birth asphyxia at term. J Pediatr 1989;114:753. 2469789 10.1016/s0022-3476(89)80132-5

[5] Boylan G , Burgoyne L , Moore C , O'Flaherty B , Rennie J . An international survey of EEG use in the neonatal intensive care unit. Acta Paediatr 2010;99:1150–5. 20353503 10.1111/j.1651-2227.2010.01809.x

[6] Klebermass K , Kuhle S , Kohlhauser‐Vollmuth C , Pollak A , Weninger M . Evaluation of the Cerebral Function Monitor as a tool for neurophysiological surveillance in neonatal intensive care patients. Childs Nerv Syst 2001;17:544–50. 11585329 10.1007/s003810100488

[7] Tao JD , Mathur AM . Using amplitude‐integrated EEG in neonatal intensive care. J Perinatol 2010;30 Suppl:S73–81. 20877412 10.1038/jp.2010.93

[8] Hellström‐Westas L , Rosén I , Swenningsen NW . Silent seizures in sick infants in early life. Diagnosis by continuous cerebral function monitoring. Acta Paediatr Scand 1985;74:741–8. 4050421 10.1111/j.1651-2227.1985.tb10024.x

[9] Gunn JK , Beca J , Hunt RW , Olischar M , Shekerdemian LS . Perioperative amplitude‐ integrated EEG and neurodevelopment in infants with congenital heart disease. Intensive Care Med 2012;38:1539–47. 22653373 10.1007/s00134-012-2608-y

[10] Meledin I , Abu Tailakh M , Gilat S , Yogev H , Golan A , Novack V et al. Comparison of Amplitude‐Integrated EEG and Conventional EEG in a Cohort of Premature Infants. Clin EEG Neurosci 2017;48:146–54. 27230038 10.1177/1550059416648044

[11] Greisen G , Hellström‐Westas L , Lou H , Rosén I , Svenningsen NW . EEG depression and germinal layer haemorrhage in the newborn. Acta Paediatr Scand 1987;76:519–25. 3604671 10.1111/j.1651-2227.1987.tb10509.x

[12] van Rooij LGM , Toet MC , van Huffelen AC , Groenendaal F , Laan W , Zecic A , et al. Effect of treatment of subclinical neonatal seizures detected with aEEG: randomized, controlled trial. Pediatrics 2010;125:e358–366. 20100767 10.1542/peds.2009-0136

[13] Jacobs SE , Berg M , Hunt R , Tarnow‐Mordi WO , Inder TE , Davis PG . Cooling for newborns with hypoxic ischaemic encephalopathy. Cochrane Database Syst Rev 2013; 2013(1):CD003311. 23440789 10.1002/14651858.CD003311.pub3 PMC7003568

[14] Edwards AD , Brocklehurst P , Gunn AJ , Halliday H , Juszczak E , Levene M , et al. Neurological outcomes at 18 months of age after moderate hypothermia for perinatal hypoxic ischaemic encephalopathy: synthesis and meta‐analysis of trial data. BMJ 2010;340:c363. 20144981 10.1136/bmj.c363 PMC2819259

[15] Ferrari F , Bedetti L , Cavalleri F , Lucaccioni L , Bertoncelli N , Guidotti I et al. Therapeutic hypothermia is associated with changes in the prognostic value of general movements. Eur J Paediatr Neurol 2023:42:53–59. 36563466 10.1016/j.ejpn.2022.12.004

[16] Gruppo di Studio di Neurologia Neonatale e Follow‐up . Raccomandazioni per l'assistenza al neonato con Encefalopatia Ipossico‐Ischemica candidato al Trattamento Ipotermico III Edizione. 2023; IdeaCPA Editor. Rome

[17] Conway JM , Walsh BH , Boylan GB , Murray DM . Mild hypoxic ischaemic encephalopathy and long term neurodevelopmental outcome ‐ A systematic review. Early Hum Dev 2018;120:80–87. 29496329 10.1016/j.earlhumdev.2018.02.007

[18] Prempunpong C , Chalak LF , Garfinkle J , Shah B , Kalra V , Rollins N , et al. Prospective research on infants with mild encephalopathy: the PRIME study. J Perinatol 2018;38:805 10.1038/jp.2017.164 PMC8592379 29095433

[19] Chalak L , Latremouille S , Mir I , Sánchez PJ , Sant'Anna G . A review of the conundrum of mild hypoxic‐ischemic encephalopathy: Current challenges and moving forward. Early Hum Dev 2018;120:88–94. 29506900 10.1016/j.earlhumdev.2018.02.008

[20] Chalak LF , Nguyen KA , Prempunpong C , Heyne R , Thayyil S , Shankaran S et al. Prospective research in infants with mild encephalopathy identified in the first six hours of life: neurodevelopmental outcomes at 18–22 months. Pediatr Res 2018;84:861–868. 30250303 10.1038/s41390-018-0174-x PMC6445543

[21] L. Dubowitz , V. Dubowitz , E. Mercuri , The Neurological Assessment of the Preterm and Full‐term Newborn Infant, second ed. Mc Keith Press, 1999.

[22] Romeo DM , Ricci D , Brogna C , Mercuri E . Use of the Hammersmith Infant Neurological Examination in infants with cerebral palsy: a critical review of the literature. Dev Med Child Neurol 2016 Mar;58:240–5. 26306473 10.1111/dmcn.12876

[23] Morgan C , Romeo DM , Chorna O , Novak I , Galea C , Del Secco S et al. The Pooled Diagnostic Accuracy of Neuroimaging, General Movements, and Neurological Examination for Diagnosing Cerebral Palsy Early in High‐Risk Infants: A Case Control Study. J Clin Med 2019;8:1879. 31694305 10.3390/jcm8111879 PMC6912336

[24] Pizzardi A , Romeo DM , Cioni M , Romeo MG , Guzzetta A . Infant neurological examination from 3 to 12 months: predictive value of the single items. Neuropediatrics 2008; 39:344–46. 19568999 10.1055/s-0029-1214423

[25] Romeo DM , Brogna C , Sini F , Romeo MG , Cota F , Ricci D . Early psychomotor development of low‐risk preterm infants: Influence of gestational age and gender. Eur J Paediatr Neurol 2016;20:518–23. 27142353 10.1016/j.ejpn.2016.04.011

[26] Green E , Stroud L , O'Connell R , Bloomfield S , Cronje J , Foxcroft C et al. Griffiths scales of child development 3rd Ed. 2016. Oxford, UK.: Hogrefe.

[27] Finder M , Boylan GB , Twomey D , Ahearne C , Murray DM , Hallberg B et al. Two‐Year Neurodevelopmental Outcomes After Mild Hypoxic Ischemic Encephalopathy in the Era of Therapeutic Hypothermia JAMA Pediatr 2020;174:48–55. 31710357 10.1001/jamapediatrics.2019.4011 PMC6865301

[28] Chalak LF , Ferriero DM , Gunn AJ , Robertson NJ , Boylan GB , Molloy EJ et al. Mild HIE and therapeutic hypothermia: gaps in knowledge with under‐powered trials. Pediatr Res 2025;97:1792–1794. 39300275 10.1038/s41390-024-03537-1 PMC12122354

[29] Singh A , Carlton C , Eiler I , Cohen S . Identifying Infants at Risk of Mild Hypoxic Ischemic Encephalopathy. Pediatr Clin North Am 2025;72:747–757. 40619197 10.1016/j.pcl.2025.03.003

[30] Montaldo, P. et al. Whole‐body hypothermia vs targeted normothermia for neonates with mild encephalopathy: a multicenter pilot randomized clinical trial. JAMA Netw. (2024). 10.1001/jamanetworkopen.2024.9119 PMC11074808 38709535

[31] Thayyil, S. & Shankaran, S. Rise and fall of therapeutic hypothermia in low resource settings: lessons from the helix trial: Authors' reply. Indian J Pediatr 2022;89:311–313. 34767188 10.1007/s12098-021-03980-6

[32] Wang Y , Xu Y , Zhou C , Cheng Y , Qiao N , Shang Q et al. Exome sequencing reveals genetic heterogeneity and clinically actionable findings in children with cerebral palsy. Nat Med 2024;30:1395–1405. 38693247 10.1038/s41591-024-02912-z

